# Soil Potassium Deficiency Reduces Cotton Fiber Strength by Accelerating and Shortening Fiber Development

**DOI:** 10.1038/srep28856

**Published:** 2016-06-28

**Authors:** Jia-Shuo Yang, Wei Hu, Wenqing Zhao, Yali Meng, Binglin Chen, Youhua Wang, Zhiguo Zhou

**Affiliations:** 1 Key Laboratory of Crop Physiology & Ecology, Department of Agronomy, College of Agriculture, Nanjing Agricultural University, Nanjing, Jiangsu Province, China

## Abstract

Low potassium (K)-induced premature senescence in cotton has been observed worldwide, but how it affects cotton fiber properties remain unclear. We hypothesized that K deficiency affects cotton fiber properties by causing disordered fiber development, which may in turn be caused by the induction of a carbohydrate acquisition difficulty. To investigate this issue, we employed a low-K-sensitive cotton cultivar *Siza 3* and a low-K-tolerant cultivar *Simian 3* and planted them in three regions of different K supply. Data concerning lint yield, *Pn* and main fiber properties were collected from three years of testing. Soil K deficiency significantly accelerated fiber cellulose accumulation and dehydration processes, which, together with previous findings, suggests that the low-K induced carbohydrate acquisition difficulty could cause disordered fiber development by stimulating the expression of functional proteins such as CDKA (cyclin-dependent kinase). As a result, fiber strength and lint weight were reduced by up to 7.8% and 2.1%, respectively. Additional quantitative analysis revealed that the degree of accelerated fiber development negatively correlated with fiber strength. According to the results of this study, it is feasible to address the effects of soil K deficiency on fiber properties using existing cultivation strategies to prevent premature senescence of cotton plants.

Premature senescence of cotton caused by potassium (K) deficiency has been observed worldwide for more than two decades^1^,^2^,^3^. This disorder significantly reduces the duration and effectiveness of cotton leaf photosynthetic capacity^2^, which may in turn reduce cotton yields by as much as 20%^4^ and could measurably impair fiber quality^5^,^6^. Intriguingly, premature senescence has also been observed in fields with relatively high levels of available K^+^, on which other crops were not affected^2^. To the best of our knowledge, this discrepancy may be caused by an imbalance between the heavy boll load and the root’s absorptive capacity of K^+2^. Additionally, high temperature stress^7^,^8^ and nitrogen deficiency^9^ could affect cotton yield and fiber properties by promoting premature senescence.

Cotton fiber is an essential raw material for the textile industry. Fiber length and strength determine yarn quality to a great extent^10^,^11^. Previous studies have confirmed that K deficiency could significantly decrease fiber length^12^,^13^, strength^14^,^15^, and micronaire value^14^. Fiber cells originate from cotton ovule epidermal cells. After rapid elongation for approximately 16 days, elongation slows, and intensive cellulose synthesis occurs until maturity. By maturity, greater than 94% of the fiber dry weight is pure cellulose^16^. The angle of cellulose deposits in the cell wall matrix^17^ and the characteristics of cellulose accumulation greatly determine the final fiber strength^18^. However, the mechanisms by which K deficiency inhibits fiber properties and fiber development remain unclear.

Most of the carbohydrate for fiber cellulose synthesis comes from the leaves subtending the cotton bolls^19^,^20^. Previous studies have confirmed that K deficiency affects cotton leaf photosynthetic capacity by inhibiting carbohydrate synthesis and the carbohydrate transportation rate^21^,^22^. Therefore, the hypothesis that K deficiency affects cotton fiber properties by inducing a carbohydrate acquisition difficulty represents a good entry point for exploring the mechanisms underlying this process.

## Results

### K Deficiency Has Negative Effects on Cotton Lint Yield and Fiber Properties

**Figure 1:**
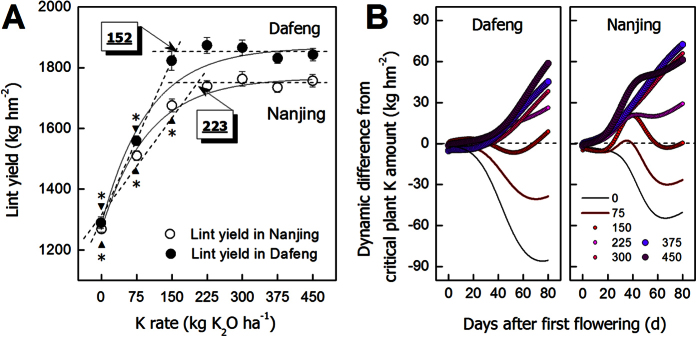
Responses of cotton lint yield to K application rates (**A**), and evaluation of cotton plant K^+^ status by modeling the dynamic change of cotton plant critical K^+^ concentration (**B**). Data (**A**,**B**) were collected in the preliminary experiment (2011), in which cotton was planted at two sites: Dafeng and Nanjing in Jiangsu Province, China. The agrotype of the Dafeng test site is coastal saline soil, with a soil soluble K^+^ concentration of 161 mg kg^−1^ before planting in 2011. Details of the Nanjing test site from 2011 to 2013 were described in the Methods. The legend in (**B**) represents the K application rate (kg K_2_O ha^−1^). Here, we showed only the data for *cv. Siza 3* as a representative.

A preliminary experiment, performed in 2011, evaluated whether K deficiency could significantly affect cotton growth. Data from both test sites showed that the K application rate had a significant impact on cotton lint yield (*cv. Siza 3*, Fig. 1A), especially when the application rate was lower than a critical value. Calculations regarding the critical application rates for achieving ideal lint yields were based on Justes *et al*.^23^. The critical rates calculated by Dafeng and Nanjing varied from 152 to 223 kg K_2_O ha^−1^. Substantial differences in agrotype and basic soluble K^+^ concentration in soil were likely responsible for this variation (see the legend of Fig. 1). The same method was also used for calculating critical cotton plant K^+^ concentrations to achieve ideal aerial biomass. After fitting all the critical K^+^ concentrations obtained across the increasing aerial biomass (15 Jul, 30 Jul, 15 Aug, 30 Aug, 15 Sep, 30 Sep and 15 Oct) using a power function, we developed a model indicating the dynamic change of cotton plant critical K^+^ concentration with respect to increasing aerial biomass. Other power functions belonging to each K treatment were also established, and the differences from the critical concentration were calculated and described in Fig. 1(B). Negative values represent K^+^ concentrations that were inadequate to support a cotton plant in achieving the ideal aerial biomass, and positive values represent K^+^ excess. Therefore, 150 kg K_2_O ha^−1^ (for Nanjing) or slightly more (for Dafeng) were the best K application strategies for cotton *cv. Siza 3* to achieve an ideal aerial biomass (Fig. 1B).

**Figure 2:**
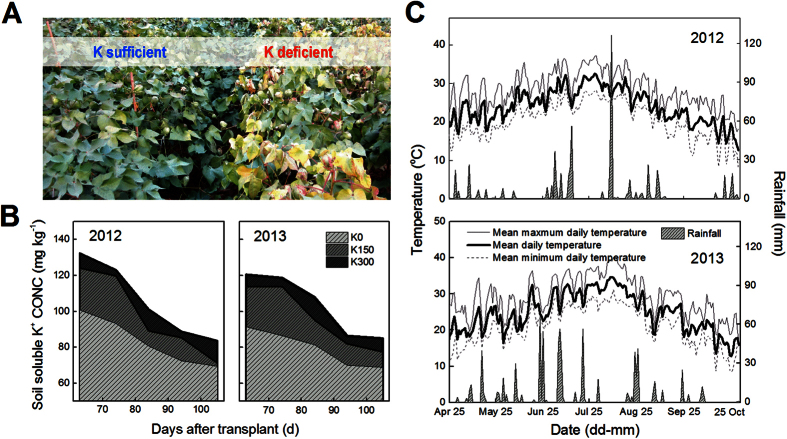
Changes in cotton plant appearance (**A**), soil soluble K^+^ concentration (**B**), and growing season temperatures and rainfall (**C**). K0, K150 and K300 represent the K application rates of 0, 150 and 300 kg K_2_O ha^−1^, respectively. As a basic condition of our experiment, the photographs of cotton plants **(A)**, the data for soil soluble K^+^ **(B)** and main climate factors **(C)** were quoted from another publication by our group (Yang *et al*.)^12^.

**Figure 3:**
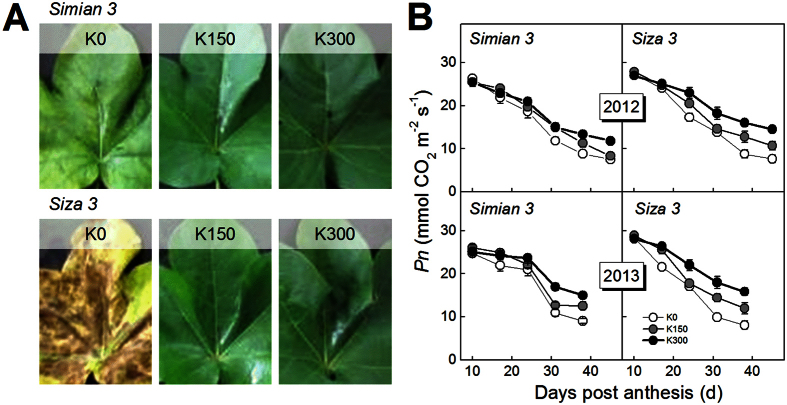
Appearance changes in cotton functional leaves (**A**) and *Pn* changes in the leaves subtending the cotton bolls on the FB_8_, the 1^st^ fruiting position (**B**). The functional leaf is the fourth fully expanded stem leaf numbered from the top and normally has the greatest contribution to the whole plant. *Pn* represents the net photosynthetic rate of the leaf. Photos in (**A**) were taken on 15 Sep. 2012. Data in (**B**) were quoted from another publication by our group (Hu *et al*.)^33^.

According to the conclusions from the preliminary 2011 experiments, a more detailed experiment was performed in Nanjing in 2012 and was repeated in 2013. These experiments involved three K application rates: 0, 150 and 300 kg K_2_O ha^−1^, corresponding to severe K deficiency, critical K supply and K sufficiency, respectively. Photos showed that K deficiency changed cotton leaf color from green to yellow and red (Fig. 2A). Soil soluble K^+^ (Fig. 2B) and main meteorological factors during the growing season (25 Apr to 1 Nov) were also recorded (Fig. 2C). In 2013, there were 43 days with mean daily temperatures (MDTs) above 30 °C, twice as many as the 21 days in 2012 (Fig. 2C). The total rainfall from 25 Apr to 1 Nov in 2013 was 720.8 mm, more than the 601.2 mm in 2012. Torrential rain occurred on 9 Aug 2012, with a rainfall of 126.3 mm. Perhaps because of the relatively higher MDT and more regular rainfall in 2013, cotton plants grew better in 2013 than in 2012. The appearance of functional leaves (the fourth fully expanded stem leaf numbered from the top) showed significant changes under conditions of K deficiency (Fig. 3A). The color of affected leaves changed to yellow and sometimes displayed disease spots when severe K deficiency (K0) affected the low-K-sensitive *cv. Siza 3*. The *Pn* (the net photosynthetic rate) of the leaves subtending the bolls (e.g., FB_8_, the 8^th^ fruiting branch, 1^st^ node) also declined significantly as a result of K deficiency, especially in older leaves (Fig. 3B). Relatively speaking, *cv. Siza 3* showed greater variations in both leaf color and *Pn* than the low-K-tolerant *cv. Simian 3*. The experiment performed in 2013 yielded similar results.

As with the results in 2011, data from 2012 and 2013 also suggested that the K application rate could significantly affect cotton lint yield (Fig. 1A and Table 1). Moreover, the data on yield components suggested that boll number was the most sensitive component affecting the response to K deficiency (CV, coefficient of variation: 5.9–16.8%), followed by boll weight (CV: 4.6–10.3%) and lint percentage (CV: 3.7–5.6%), with relatively stable effects (Table 1). According to the consistent inhibition of K deficiency on lint percentage over three years, it appeared that K deficiency had a greater impact on fiber development than on seed, at least in terms of biomass.

Generally, both cultivars tested would have 14–16 FBs (fruiting branches) before artificial topping in Nanjing, China, where they were sown at the end of April and transplanted at the end of May. Accordingly, the 3^rd^, 8^th^ and 13^th^ FBs were selected as representatives of the bottom, middle and top parts, respectively, of the cotton plant. The properties (length, strength and micronaire) of fibers grown on the FB_8_ were measured (Table 2). Significant differences in both fiber length and strength existed among the three conditions of K supply, especially for the years 2012 and 2013. However, the differences in fiber micronaire were not as significant (Table 2). CV analysis revealed similar tendencies, in which fiber length (up to 7.5%) varied more than fiber strength (up to 4.1%), and much more than fiber micronaire (up to 3.5%) (Table 2).

**Figure 4:**
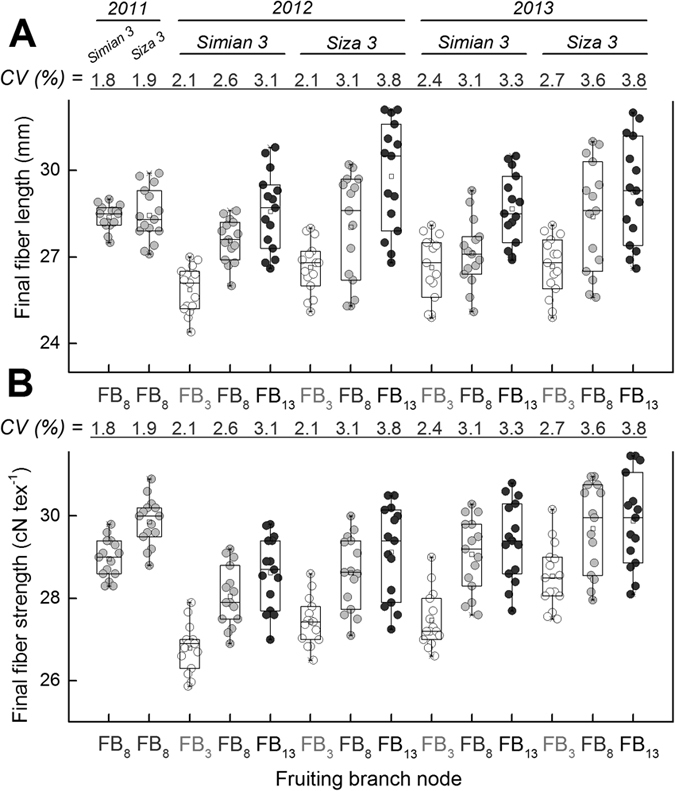
Variations in mature fiber length (**A**) and strength (**B**) under varying conditions of K supplementation. Fiber samples were picked one week after boll opening. For the purpose of detecting the effects of K supplementation on fiber length **(A)** and strength **(B)**, data from three K conditions were compared to demonstrate that K300 tended to have higher fiber strength and K0 tended to have lower fiber strength. Each FB position had five biological samples for each K condition. In the box-and-whisker plots, the bottom, middle and top lines indicate the first, second and third quartiles; the ends of the whiskers represent the data minimum and maximum values.

After excluding fiber micronaire because of its minor response to K deficiency, we plotted detailed data point (five replicates) of fiber length (Fig. 4A) and fiber strength (Fig. 4B). The middle (8^th^) and higher (13^th^) FBs tended to yield longer and stronger cotton fibers that were more sensitive to impairment when suffering from severe K deficiency (K0). Both FB_8_ and FB_13_ were sufficiently sensitive to indicate the influence of K deficiency on fiber development. However, FB_8_ had the advantages of being more representative and stable. Therefore, we used FB_8_ to perform a detailed study of the response of developing fibers to K deficiency.

**Figure 5:**
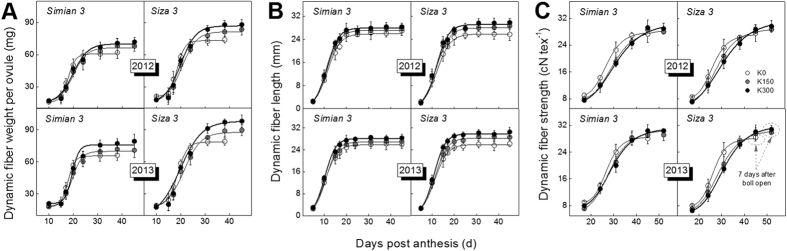
Dynamic changes in fiber weight per ovule (dry weight, **A**), fiber length (**B**) and fiber strength (**C**) by days post anthesis under three K supplements on the FB_8_.

The changes in fiber dry weight (Fig. 5A), fiber length (Fig. 5B) and fiber strength (Fig. 5C) with days post anthesis (DPA) were fitted using equations (1), (2) and (3) in Methods, respectively. As seen in Fig. 5, the difference in fiber dry weight and strength were likely due to the earlier termination of growth. The patterns of K0 occurred ahead of those of K300 for fiber dry weight and fiber strength, but not for fiber length. A comparable phenomenon was observed in the BMP (cotton boll maturation period) on FB_8_, which was generally 39/38 days (2012/2013) for K0, 42/41 days (2012/2013) for K150 and 44/42 days (2012/2013) for K300. We speculate that K deficiency could accelerate the developmental process in not only cotton leaves and bolls but also cotton fibers. This acceleration phenomenon could be a new explanation for the effects of K deficiency on fiber strength. However, the reduction of fiber length appeared to occur as a result of osmoregulation rather than developmental acceleration^12^.

### Quantification of Cotton Fiber Physiological Age and its Relationship with Fiber Strength

**Figure 6:**
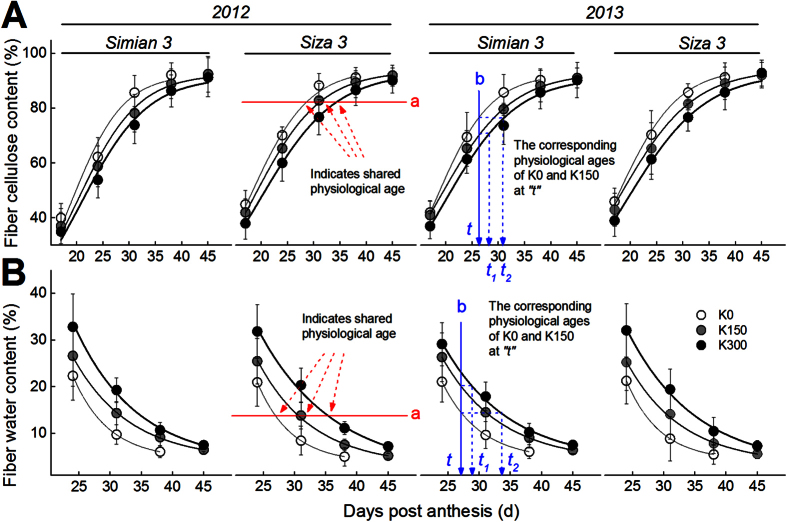
Changes in fiber cellulose content (**A**) and fiber water content (**B**) during fiber development under three K supplement conditions on FB_8_. Straight lines “a” and “b” are guide lines for defining and calculating fiber physiological age, respectively.

**Figure 7:**
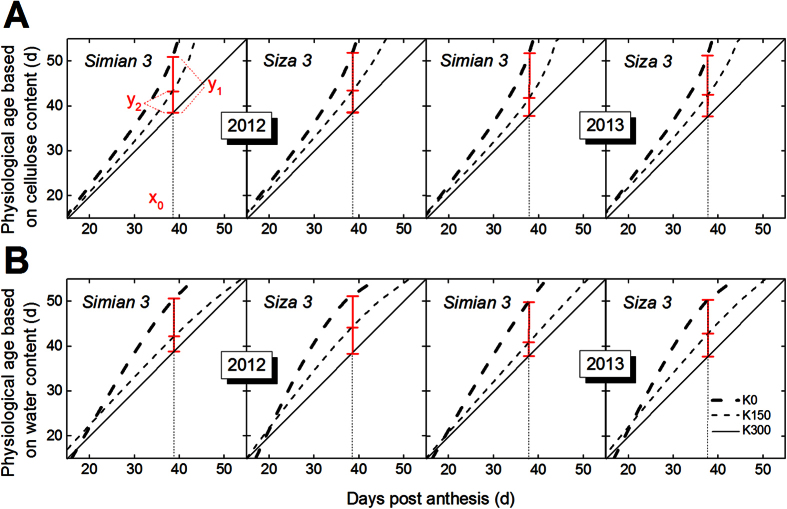
Advanced fiber physiological age under conditions of K deficiency (K0 and K150) compared with K sufficiency (K300) as defined by cellulose accumulation (**A**) and dehydration (**B**) patterns. “x_0_” was the natural age when bolls of K0 opened (39 and 38 DPA in 2012 and 2013, respectively). Line segments “y_1_” and “y_2_” demonstrate the fiber physiological age gaps from K0 to K300 and from K150 to K300, respectively at the time point “x_0_”. The results were calculated based on the fibers grown on the FB_8_.

The changes in fiber cellulose content (Fig. 6A) and water content (Fig. 6B) during fiber development were fitted with the sigmoid equation (4) and the exponential equation (5) in Methods, respectively. There were small differences in the effects of different levels of K supply on the final values of both fiber cellulose and water content. Therefore, the difference in K treatments during fiber development could be used to indicate the delay or acceleration caused by K treatments at that time. For the current study, we defined K0 and K150 to have the same physiological age as K300 when they shared the same Y value (see the horizontal line “a” in Fig. 6A,B). Based on this definition, K0 and K150 could reach the same physiological age as K300 did at an earlier DPA. Correspondingly, at time point “t”, the physiological ages of K0 and K150 were, respectively, “t_1_” and “t_2_” (see the vertical line “b” in Fig. 6). Thereafter, the vertical line “b” was moved gradually from 15 DPA to 55 DPA, and the dynamic changes in physiological age gaps, “t_1_-t” and “t_2_-t” (Fig. 6A,B), were drawn in Fig. 7. The Y value in Fig. 7 represents the physiological age, and “y_1_” and “y_2_” are the advanced physiological ages of K0 and K150 compared with K300. For example, the physiological age of K300 at “x_0_” was “x_0_”, but for K0 and K150, they were “x_0_+y_1_” and “x_0_+y_2_”, respectively. These gaps increased with DPA when they were computed based on fiber cellulose accumulation (Fig. 7A), but kept increasing until only approximately 38 DPA when they were computed based on fiber dehydration (Fig. 7B). The possible causes for this difference might be that cotton bolls grown on the K0 plants opened earlier (at 39 DPA in 2012 and at 38 DPA in 2013) and stopped growing afterwards, whereas those on the K150 and K300 plants opened much later (at 42/41 DPA in 2012/2013 for K150 plants and at 44/42 DPA in 2012/2013 for K300 plants). Therefore, the result/pattern after 39/38 DPA (2012/2013) in Fig. 7 was not reliable, and the following analysis was set at the time when the cotton bolls of K0 plants opened.

**Figure 8:**
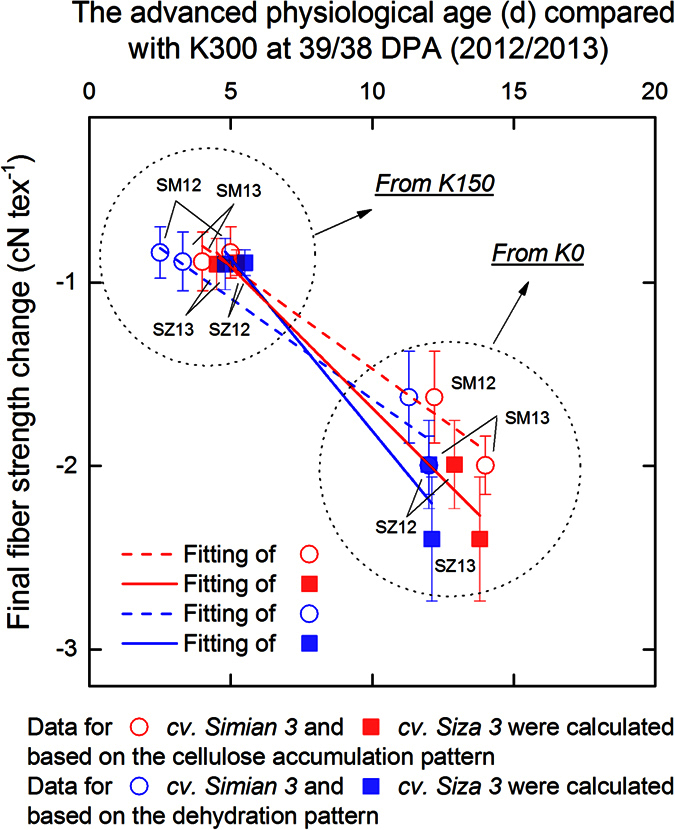
Correlation between advanced physiological age at the time when the K0 boll opened (39 days for 2012, and 38 days for 2013) and fiber strength reduction on the FB_8_. SM12, SZ12, SM13 and SZ13 represent the data for *cv. Simian 3* in 2012, *cv. Siza 3* in 2012, *cv. Simian 3* in 2013, and *cv. Siza 3* in 2013, respectively.

The advanced physiological age compared with K300 at 39/38 DPA (2012/2013) had a negative impact on fiber strength; this impact was greater on *cv. Siza 3* than on *cv. Simian 3* (see the slopes of the regression lines in Fig. 8). Compared with K0, K150 had less impact on fiber physiological age. Calculations based on different physiological indices (e.g., fiber cellulose accumulation and fiber dehydration) revealed the same result. Interestingly, from Figs 7 and 8, we observed that the physiological age gap between K treatments could reach 10–15 days. However, the actual difference on BMP reached only 4–5 days (mentioned above). A possible explanation for this result is that the fiber physiological age gap is completely different from BMP. BMP has a direct relationship with capsule wall maturity, but not with fiber maturity. For example, when a cotton boll opens, its BMP is fixed, whereas the fiber properties continue to change^24^. This relationship helps establish a connection between the low-K-induced acceleration of fiber development and the corresponding reduction of fiber strength.

## Discussion

**Figure 9:**
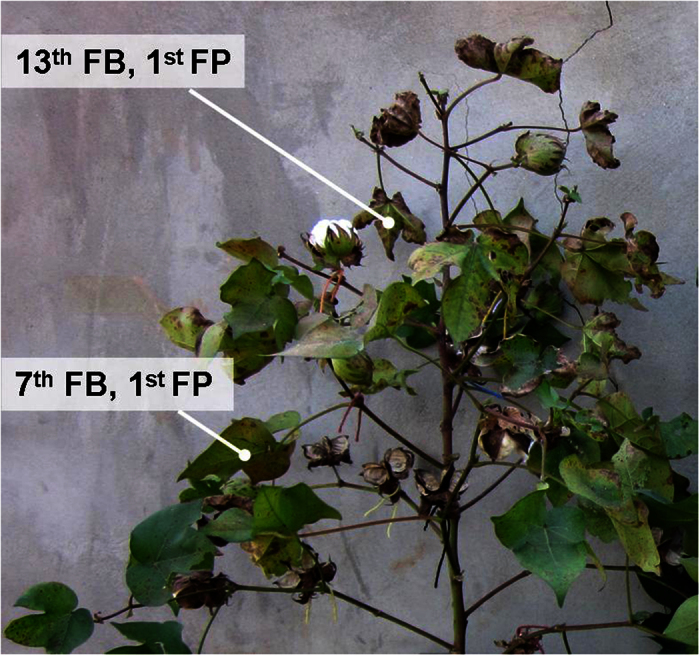
Differences in K deficiency between young and old leaves, both of which were growing at the 1^st^ FP (fruiting position).

There are two types of K-deficient disorders relevant to cotton. In type I, K deficiency syndrome first affects older leaves, which is called the classical K deficiency. This disorder usually occurs on soil with strongly fixed K^1^ or inherently K-deficient soil^25^. Type II is called K deficiency-induced premature senescence, in which K-deficient signs appear on younger rather than older leaves^26^, and which predominantly occurs on soil with a relatively high levels of available K^2^. Compared with type I, the type II disorder is caused by an intrinsic imbalance between K^+^ supply and K^+^ demand, which commonly occurs in conditions of relatively high boll load^2^,^26^. For the current study, we suspect type II K deficiency because of the large number of specks found on younger leaves (Fig. 9) and the greater reduction of fiber strength among late-season bolls (Fig. 4). However, there was an apparent difference between previous studies and ours in that boll loads of K-deficient plants in our experiment were not larger than those of the controls. Specifically, the boll loads of severely K-deficient plants in our study (K0) were 8.5, 15.6 and 22.2% (2011–2013, *cv. Simian 3*) smaller, or 12.4, 12.6 and 28.7% (2011–2013, *cv. Siza 3*) smaller than in the K-sufficient plants (K300, Table 1). Therefore, reasons other than boll load could have caused the premature senescence observed in this study. Potential causes could include environmental factors, endogenous hormones, disease, mineral element imbalances or soil construction^22^.

A number of studies have indicated that severe K deficiency could significantly reduce cotton leaf photosynthetic capacity^27^,^28^ and reduce phloem loading speeds^29^,^30^. As 60–87% of the total photoassimilate for cotton boll development comes from the subtending leaves^20^,^31^, a strong connection between carbohydrate starvation^32^,^33^ and accelerated fiber development exists. In fact, previous studies have already shown that plant carbohydrate supply barriers could induce premature development of many organs^34^. Based on our results, we determined that the carbohydrate supply barrier could also lead to acceleration of fiber development (Figs 5 and 6).

The reduction of fiber strength in our research (Fig. 8) is potentially caused by accelerated fiber development (Fig. 5B), which was presumably induced by the carbohydrate acquisition difficulty^33^,^34^. Studies have indicated that CDKA (cyclin-dependent kinase, also known as kinase p34^cdc2^) could regulate plant cell cycles^34^,^35^ and that difficulty obtaining sufficient carbohydrates for plant development could stimulate the generation of CDKA^34^. Previous research has reported a positive correlation between CDKA activity and the cell growth rate in Arabidopsis roots^35^ as a result of comparing 18 ecotypes. Similar results have also been found among maize leaf cells suffering from water stress^34^. Therefore, CDKA was a potential factor affecting cotton fiber cells and was activated by the low-K-induced carbohydrate acquisition difficulty, which reduced fiber strength by accelerating fiber development.

Carbohydrate acquisition difficulty could also be a cause of reductions in cotton lint yield. A recent study of cotton demonstrated a positive correlation between boll weight and sucrose transport capacity from leaf to boll^20^. As a result, the carbohydrate acquisition difficulty induced by K deficiency^36^,^37^,^38^ could lead to cotton yield reductions of as much as 20%^4^. An interesting phenomenon observed in the current study was that the effect of different K conditions on fiber dry weight accumulation (Fig. 5A) was very similar to the effect on fiber strength formation (Fig. 5C), in that the K0 conditions resulted in earlier changes than the other conditions. We speculate that K deficiency might inhibit cotton lint yield in terms of fiber weight per ovule (Fig. 5A) through a pathway similar to the one affecting fiber strength. This point is worthy of further investigation.

Two hypotheses have been proposed to explain why some cotton varieties are more prone to premature senescence: (1) relatively higher boll loads and higher yield potential require amounts of K and P that their root systems cannot provide^26^,^39^; and (2) the lower net *P*_*n*_ and carbon assimilation, along with higher nitrogen assimilation and reactive oxygen species (ROS) accumulation in the leaves, make them more sensitive to K deficiency^40^. The results from the current study (Fig. 8) showed that *cv. Siza 3* exhibited a greater reduction of fiber strength than *cv. Simian* 3, despite similarly advanced physiological age (K0). According to our investigations of boll load (Table 1) and leaf *Pn* (Fig. 3), the latter hypothesis seems more appropriate, which means that metabolic rather than supply features are more strongly related to the low-K-induced acceleration of fiber development. Previous studies showed that some cotton cultivars could achieve the same biomass as others under K-sufficient conditions, but not under K-deficient conditions^40^,^41^. Data from our research similarly found that the low-K-sensitive *cv. Siza 3* had consistently greater CV of lint yield than the low-K-tolerant *cv. Simian 3* (Table 1). These results suggest that the low-K-sensitive cultivars are prone to show sensibility only under K-deficient conditions.

Up to this point, a series of field management strategies has been established to address the premature senescence caused by K deficiency. These strategies have included features such as reducing the occurrence of waterlogging by employing more appropriate irrigation and field layout schemes^39^, minimizing soil compaction by conducting tillage operations^35^, and implementing a late planting production system (LPPS)^42^. The significance of our current study is that we have established a physiological connection between the low-K-induced carbohydrate acquisition difficulty and fiber strength and have made it easy and feasible to cope with the inhibition on fiber development using strategies already employed to defend against cotton plant senescence.

## Methods

### Test Site Description, Experimental Design and Crop Management

The low-K-tolerant cotton cultivar *Simian 3* and low-K-sensitive cotton cultivar *Siza 3* were obtained by comparing 12 ecotypes (*Gossypium hirsutum* L.)^43^ for study. Cluster analysis was used to classify these ecotypes by considering boll number, lint weight per boll, fiber length and fiber strength. Afterwards, *cv. Simian 3* and *cv. Siza 3* were planted in a purpose-built test field (yellow-brown loam) at the Pailou test site (Nanjing Agricultural University, Jiangsu, China) for three years (2011, 2012 and 2013), with the constant density of 33,600 plants ha^−1^. The 2011 planting was a preliminary experiment, in which the soil soluble K concentration (0–40 cm) before planting was 115 mg kg^−1^, not consistent with severe K deficiency. Accordingly, limited data of cotton fiber properties in 2011 was obtained. However, the plantings in 2012 and 2013 were successfully prepared with regular K depletion before each year’s planting. One season of wheat and one season of peas were planted for K depletion. The soil soluble K concentrations (0–40 cm) before planting in 2012 and 2013 were 92 and 86 mg kg^−1^, respectively. There were three individual fields for each year’s experiment, which eliminated fertilizer residual effects.

K treatments of 0, 150 and 300 kg K_2_O ha^−1^ (K0, K150 and K300) were arranged in a randomized block design with 3 field replicates. The K0 treatment combined with K-deficient soil created severely K-deficient conditions for cotton growth, whereas the 150 and 300 kg K_2_O ha^−1^ conditions induced mildly K-deficient and K-sufficient conditions, respectively^36^,^44^. Each test plot was 6.6 × 13 meters with 16 rows. K fertilizer (potassium sulfate, in which K_2_O content is 50%) was applied to holes at the start of cotton flowering. N fertilization (240 kg N ha^−1^), P fertilization (120 kg P_2_O_5_ ha^−1^), irrigation and pest control were performed in accordance with recommended practices. No symptoms indicating inappropriate N supply, water stress or pest pressure negatively influenced cotton growth.

### Soil Soluble K^+^, Plant K^+^ Concentration, and Fiber Length, Strength, Micronaire, Dry Weight and Water Content Measurements

Soil was sampled from nine locations (0–40 cm) for each plot. Samples were mixed and dried naturally in the air to constant weight and were then ground into powder for use. Soil samples were collected on 25 Jul, 5 Aug, 15 Aug, 25 Aug and 5 Sep of 2012 and 2013, corresponding to 63, 74, 84, 94 and 105 days after transplant (22 May) for both years. For the reason mentioned above, the 2011 dataset was a preliminary experiment, did not include dynamic soil soluble K^+^ data. Soil soluble K^+^ was determined following the method described in Yang *et al*. 12. Cotton plant samples (15 Jul, 30 Jul, 15 Aug, 30 Aug, 15 Sep, 30 Sep and 15 Oct of 2011) were detached and then digested with H_2_SO_4_-H_2_O_2_^45^, after which the K^+^ concentrations were measured using the same procedure as soil soluble K^+^ concentration measurements^12^.

Cotton boll age was determined by tagging the fully opened white flower (0 DPA). The first fruit-bearing sympodial branch was defined as the 1^st^ FB. Generally, according to each plant’s growth habits and the climate in Nanjing, China, the two cultivars would have 14 to 16 FBs before artificial topping. Therefore, the 3^rd^, 8^th^ and 13^th^ FBs were tagged as representatives of the lower, middle and upper FBs, respectively, of the cotton plant. For mature fiber sampling, cotton fibers grown on each FB were picked one week after boll opening; for immature sampling, 15, 17, 20, 24, 31, 38 and 45 DPA samples of bolls grown on the FB_8_ were collected.

Mature fiber properties, including fiber length, strength and micronaire, were determined using the cotton fiber quality measurement system USTER HVI MF100 (Uster Technologies, Switzerland). The measurement was performed in a standard testing room with constant temperature (20 ± 2 °C) and humidity (65 ± 2%). Fiber samples were placed in this room for 48 h before measurement.

The length measurement of immature fibers was based on Yang *et al*.^12^. Prior to the strength measurement of immature fibers, they were preprocessed in an oven dryer at 60 °C for 0.5 h and then at 40 °C for 2 days; they were then placed into a standard testing room with constant temperature (20 ± 2 °C) and humidity (65 ± 2%) for 48 h. A Functional Fiber-bundle Tensile Tester (KX-154, Shanghai Kangxin Photoelectric Instrument Co., Ltd., China) was used for the strength measurement of immature fibers.

Fresh fiber water content was calculated from the difference in weight before and after the drying procedure.

### Fiber Cellulose Concentration Analyses

Fibers recycled from strength measurements were digested using acetic-nitric acid. The determination of cellulose concentration was based on the anthrone colorimetry method^46^.

### Statistical Analyses

Figures 1, 2, 3, 4, 5, 6, 7, 8 were drawn using Origin 9.0 (OriginLab, Northampton, Massachusetts, USA); Fig. 9 was drawn using PowerPoint 2003 (Microsoft).

The dynamic changes of fiber dry weight per ovule, fiber length, fiber strength and fiber cellulose concentration were fitted using the following logistic equations (1), (2), (3) and (4), respectively^11^,^12^. In all equations, “*t*” represents days post anthesis (DPA).


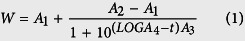


“*W*” represents fiber dry weight. “*A*_*1*_*, A*_*2*_*, A*_*3*_*, A*_*4*_” are parameters.


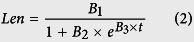


*“Len”* represents fiber length. “*B*_*1*_, *B*_*2*_, *B*_*3*_” are parameters.


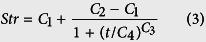


“*Str*” represents fiber length, “*C*_*1*_*, C*_*2*_*, C*_*3*_*, C*_*4*_” are parameters.


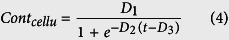


“*Cont*_*cellu*_” represents fiber cellulose content, “*D*_*1*_*, D*_*2*_*, D*_*3*_” are parameters.

The dynamic change of fiber water content was regressed by the exponential equation (5), in which “*Cont*_*water*_” represents fiber water content and “*E*_*1*_*, E*_*2*_*, E*_*3*_” are parameters.


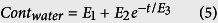


## Additional Information

**How to cite this article**: Yang, J.-S. *et al*. Soil Potassium Deficiency Reduces Cotton Fiber Strength by Accelerating and Shortening Fiber Development. *Sci. Rep.* **6**, 28856; doi: 10.1038/srep28856 (2016).

## Figures and Tables

**Table 1:** Effects of three K application rates on cotton lint yield and lint yield components (boll number, boll weight and lint percentage).

K rate	2011		2012		2013	
	*Simian 3*	*Siza 3*	*Simian 3*	*Siza 3*	*Simian 3*	*Siza 3*
*Boll number* (10^4^ ha^−1^)						
K0	74.9 b	67.6 b	81.4 c	74.3 c	67.1 c	50.8 c
K150	73.5 b	78.8 a	85.7 b	82.5 b	73.1 b	60.2 b
K300	81.9 a	77.2 a	96.4 a	85.0 a	86.2 a	71.2 a
CV (%)	5.9	8.1	8.8	6.9	12.9	16.8
*Boll weight* (g)						
K0	4.7 b	4.9 b	4.1 b	4.4 c	4.3 b	5.2 b
K150	5.1 a	5.4 a	4.4 ab	5.1 b	5.1 a	6.0 a
K300	5.1 a	5.5 a	4.6 a	5.4 a	4.9 a	6.1 a
CV (%)	4.6	6.1	5.8	10.3	8.7	8.6
*Lint percentage* (%)						
K0	36.4 b	38.3 b	34.1 b	36.4 c	37.2 c	42.3 b
K150	38.3 a	39.4 b	36.4 a	39.2 b	39.2 b	45.0 a
K300	39.1 a	41.5 a	37.0 a	40.7 a	40.8 a	45.9 a
CV (%)	3.7	4.1	4.3	5.6	4.6	4.2
*Lint yield* (kg ha^−1^)						
K0	1281 c	1269 b	1138 c	1190 c	1073 c	1118 c
K150	1436 b	1676 a	1373 b	1650 b	1461 b	1625 b
K300	1633 a	1763 a	1640 a	1868 a	1724 a	1994 a
CV (%)	12.2	16.8	18.2	22.1	23.1	27.9

CV, the coefficient of variation. Data followed by different letters (a, b, c) in one group indicate statistically significant differences at the p < 0.05 level based on ANOVA. The results show the mean values of 9 samples for boll number; 15 samples for boll weight; 15 samples for lint percentage; and 3 samples for lint yield.

**Table 2:** Changes in main cotton fiber properties (length, strength and micronaire) of the FB_8_ when grown in three K application rates.

K rate	2011		2012		2013	
	*Simian 3*	*Siza 3*	*Simian 3*	*Siza 3*	*Simian 3*	*Siza 3*
*Fiber length* (mm)						
K0	28.0 a	27.5 c	26.9 c	25.7 c	26.0 c	26.2 c
K150	28.4 a	28.3 b	27.6 b	28.6 b	27.0 b	28.6 b
K300	28.7 a	29.5 a	28.3 a	29.8 a	28.3 a	30.5 a
CV (%)	1.2	3.5	2.6	7.5	4.3	7.5
*Fiber strength* (cN tex^−1^)						
K0	28.4 b	29.2 c	27.3 c	27.6 c	28.0 c	28.4 c
K150	29.0 ab	29.9 b	28.0 b	28.7 b	29.2 b	29.9 b
K300	29.5 a	30.4 a	28.9 a	29.6 a	30.0 a	30.8 a
CV (%)	1.8	2.0	2.9	3.5	3.5	4.1
*Fiber micronaire*						
K0	4.8 a	4.7 a	5.2 a	4.9 a	4.2 b	5.1 ab
K150	4.9 a	4.8 a	5.1 a	4.8 a	4.4 ab	5.2 a
K300	4.9 a	4.7 a	5.3 a	5.0 a	4.5 a	4.9 b
CV (%)	1.0	1.7	1.9	2.0	3.5	3.0

Data followed by different letters (a, b, c) in one group indicate statistically significant differences at the p < 0.05 level based on ANOVA. The fiber properties above were measured from the FB_8_. Each number was the mean value of 5 samples.
